# Publics’ Perceptions of Community Pharmacists and Satisfaction with Pharmacy Services in Al-Madinah City, Saudi Arabia: A Cross Sectional Study

**DOI:** 10.3390/medicina58030432

**Published:** 2022-03-16

**Authors:** Amal A. El-Kholy, Khaled Abdelaal, Hussain Alqhtani, Basel A. Abdel-Wahab, Mohamed M. M. Abdel-Latif

**Affiliations:** 1 Department of Clinical Pharmacy, Faculty of Pharmacy, Ain Shams University, Cairo 11566, Egypt; 2 EPCRS Excellence Center, Plant Pathology and Biotechnology Lab., Faculty of Agriculture, Kafrelsheikh University, Kafrelsheikh 33516, Egypt; khaled.elhaies@gmail.com; 3 Department of Clinical Pharmacy, College of Pharmacy, Najran University, Najran 1988, Saudi Arabia; 4 Department of Pharmacology, College of Pharmacy, Najran University, Najran 1988, Saudi Arabia; baselabdelwahab@gmail.com; 5 Department of Pharmacology, College of Medicine, Assiut University, Assiut 71111, Egypt; 6 Department of Clinical Pharmacy, Faculty of Pharmacy, Assiut University, Assiut 56123, Egypt; mohamedabdelatif48@gmail.com

**Keywords:** public, community pharmacist, pharmacy services, satisfaction, Saudi Arabia

## Abstract

*Background and Objectives*: Pharmacists play a major role in serving patients and delivering pharmaceutical services to the community. It is unclear whether the public fully appreciates what pharmacists can do as key health care providers. This study aims to examine public perceptions of community pharmacists and levels of satisfaction with pharmacy services. *Materials and Methods*: A cross-sectional study was conducted on a randomly selected sample population (*n* = 1000) in Saudi Arabia over a period of six months from January through June 2019. A 40-item, structured, self-administered questionnaire was used, comprised of questions on the demographics characteristics of the respondents and their satisfaction with pharmacy services. Descriptive statistics were used to analyze the data. *Results*: The response rate of the survey was 76.92%. Public opinions were influenced by pharmacists’ availability and knowledge, service promptness, and counseling services. Overall, 80.5% of respondents agreed that community pharmacists treat them with respect. Doctors were identified as the preferred source of drug therapy consultation by 58.7% and pharmacists by 41.29%. About 72.8% of respondents agreed that pharmacists provided them with clear instructions about medication use, and 70.2% trusted pharmacists’ opinions about medications. About 62.5% of respondents expressed satisfaction with pharmacists, and 64.8% with pharmacy services. *Conclusions*: Customers’ opinions were influenced by pharmacists’ availability and knowledge, pharmacy service promptness, pharmacy location, waiting area, medication knowledge, and counseling. However, the public was greatly satisfied with community pharmacists’ professionalism and pharmaceutical services. This positive perception provides an opportunity for pharmacists to extend their roles as healthcare professionals.

## 1. Introduction

In recent years, pharmacists have made great efforts to shift their focus from medication dispensing to patient care. The field has been undergoing a paradigm shift in this respect, from product-oriented functions, i.e., dispensing and compounding medications, to the provision of pharmaceutical services, information, and pharmaceutical care [1].

Pharmaceutical care contributes to reducing drug-related morbidities and mortalities, improving clinical outcomes and health-related quality of life, and lowering medical costs [2,3,4]. The cornerstone of pharmaceutical care success is the quality of patient–pharmacist relationships. In such relationships, patients grant authority to pharmacists to manage their health and well-being. In turn, pharmacists accept responsibility to take care of the well-being of the patients [2]. Community pharmacies are the front doors of medical advice and the points of sale of pharmaceutical products. Customer loyalty is crucial in the medical and pharmacy business [5].

Problems in pharmacist consultation can occur when patients and pharmacists have different expectations regarding the pharmacist’s role and the provision of services. Patients who have low expectations regarding a consultation with a pharmacist receive fewer consultations than those with higher expectations [6]. Therefore, the patient–pharmacist relationship is largely influenced by the level of the patient’s trust in the pharmacist [7]. Trust in pharmacists could be defined as “patients’ willingness to be vulnerable to the actions of pharmacists based on the expectation that pharmacists will do what is best for patients, irrespective of patients’ ability to monitor pharmacists” [8].

Patient satisfaction is an important indicator of the quality of health care services, and an important predictor of the relationship with the health care provider and of adhering to a medication regimen [9]. However, it is difficult to identify a single factor that is directly associated with a low or high level of patient satisfaction with the provided health care services. Patient satisfaction has been conceptualized differently over the last 15 years, and has been characterized as a complex construct [10,11,12]. A variety of factors might be involved in patient satisfaction degree. Some of these are patient demographics, health status, characteristics of the health care provider, such as technical expertise, interest in patient-oriented care, and waiting time [13,14]. Furthermore, patient satisfaction is found to be directly associated with patient expectations; patient satisfaction can be defined as the sum of patient expectations and perceptions of the treatment or pharmaceutical service provided to them [13,14,15]. Implementing changes based on this feedback is vital for upgrading the health system and attaining optimal patient satisfaction [16]. This interaction between patients and pharmacists has been thoroughly assessed in many developed countries [17,18,19,20]. However, this research is often not generalized to developing countries such as those in the Middle East, where the priority is still traditional pharmacy practices [21]. There have been some efforts to gauge patient satisfaction in some Middle East countries [22,23,24]. Al-Arifi [25] reported an improvement in the image and professional performance of community pharmacists in Saudi Arabia. Saudi patients showed increased satisfaction, perception, and appreciation of the role of pharmacists in health care, as about 38% of respondents assured suo moto counseling by the pharmacist, 35% reported that pharmacists play an active role in their compliance to treatments, 43% acknowledged the role of pharmacists in solving medication related problems, 34% considered pharmacists as health awareness providers and 44.6% felt that pharmacists are indispensable and an effective part of the health care system.

This study’s objectives are to assess the public view of the role of community pharmacists in delivering pharmacy services. In addition, we aimed to determine the public’s opinions of, and satisfaction with, the community pharmacy services currently provided in Al-Madinah Al-Munawwarah, Saudi Arabia.

## 2. Materials and Methods

### 2.1. Study Design

A cross-sectional study was conducted over a period of six months from January through June 2019 in Al-Madinah Al-Munawwarah, Saudi Arabia. The study was approved by the Ethical Committee of Taibah University.

### 2.2. Development and Validation of the Questionnaire

A self-administered questionnaire was developed after an extensive literature review [25,26,27,28,29,30]. The survey was composed of five sections, each of which comprised a series of questions regarding views among members of the public of community pharmacists (general image of pharmacists, their availability, politeness, respect, trust, knowledge, advice, and counseling), an evaluation of pharmacy services (promptness of service, reason and frequency of visits, waiting time, screening and monitoring tests, and buying medication), an evaluation of community pharmacies (appearance, availability of medicines, use of a computerized system, waiting area, and privacy), barriers to communication with community pharmacists, and satisfaction with the role of the pharmacists and pharmacy services. In addition, questions regarding demographic characteristics (age, gender, nationality, education, marital status, health status, employment, and income) were included. The questionnaire was initially developed in English and reviewed by the head of clinical pharmacy to ensure the appropriateness and validity of the content. Then, it was translated into Arabic by a linguistic expert and checked by another academic in the department of clinical pharmacy who is a content expert in the subject of the present study. The survey was tested with 50 members from the target population for its comprehensibility, clarity and for validation of the translation. The questionnaire was designed to be administered during a face-to-face interview and was typically completed in 10 min.

### 2.3. Study Participants

A total of 100 community pharmacies in Al-Madinah city were randomly selected for visits according to their geographical distribution. They represent about 20% of all community pharmacies in the city. The selection of pharmacies was done at random with a clear intention to include different areas of Al-Madinah. Permission from pharmacists to approach patients when they entered the pharmacy was requested and obtained. All Arabic-speaking consumers above 18 years of age from both genders who responded to the questionnaire were interviewed by an assigned pharmacist. The study’s objective was described to them, and they were invited to complete the questionnaire after signing the consent form. The participants were assured that the information derived from the questionnaires will be kept confidential and the data will be presented as groups. The sample size of the study was calculated by referring to similar studies [25,26,27,28,29,30]. A total of 1000 out of 1300 participants were enrolled.

### 2.4. Data Collection and Analysis

Completed questionnaires were collected from all participants to evaluate their responses. Data were analyzed using the Statistical Package for the Social Sciences (SPSS), version 20 (IBM, SPSS, Chicago, IL, USA). In this questionnaire, data are presented in percentages (%). Descriptive statistics including mean and percent were used for statistical analyses. The results were presented as numbers with percentages or graphic representations for categorical variables. Incomplete questionnaires were excluded from the study.

## 3. Results

### 3.1. Demographic Characteristics of the Respondents

A total of 1000 participants agreed to take part in this study out of the 1300 people approached, indicating a response rate of 76.92%. A total of 48.9% of respondents fell in the 20–39 age group. Most respondents (57.9%) were college graduates or higher degree holders. The education level of participants was shifted toward the highly educated in comparison to the general population. Of the respondents, 51.6% were employed with a reasonable income. Nearly one half of respondents (49.7%) were single, and 44.3% were married. The ratio of Saudi to non-Saudi individuals was 90.5:9.5. The respondents had a mix of health problems, but diabetes mellitus, hypertension, and asthma were the most prevalent among them. Details of the demographic data of the respondents are shown in Table 1.

### 3.2. Customers’ Views of the Pharmacy Facility, Accessibility, and Privacy

The respondents were asked to assess their preferred pharmacy’s amenities, accessibility, and services, with one of the following responses: poor, good, fair, very good to excellent. The survey included questions on the appearance, availability of medicines and computerized systems, and waiting area at the pharmacy. Details of the participants’ responses are shown in Table 2. About 49% of respondents were satisfied the availability of medicines at the pharmacy. Most respondents (74.6%) stated that pharmacists were always available to serve them. However, 63.9% of respondents mentioned that there was no dedicated area available in the pharmacy where they could speak with the pharmacist without being overheard. Regarding recognition of the pharmacist by nonpharmacist staff in the pharmacy, most respondents (71.5%) stated they could easily distinguish the pharmacist from other staff members. A total of 717 (71.7%) respondents stated that the community pharmacy had a computer system.

### 3.3. Views of Customers on Pharmacy Appearance, Waiting Area, and Busyness

**Figure 1:**
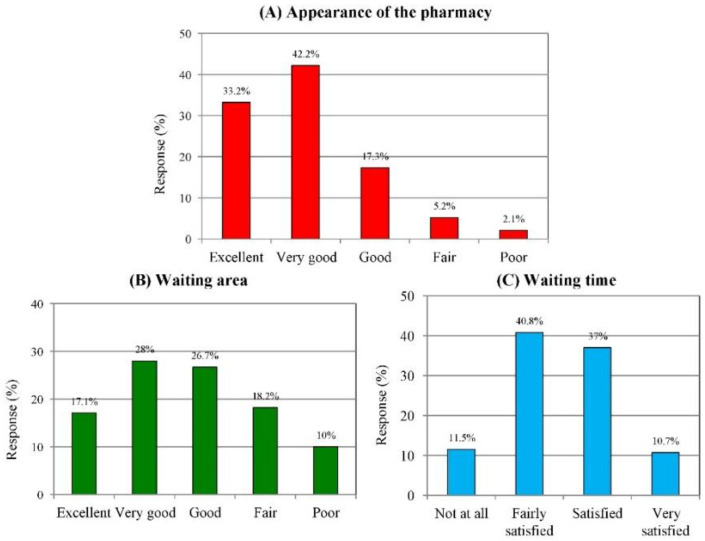
Customers’ views on pharmacy appearance (**A**), waiting area (**B**) and busyness (**C**).

There was general satisfaction with the appearance and cleanliness of the pharmacies; 33.2% rated this parameter as excellent, 42.2% as very good, 17.3% as good, 5.2% as fair, and 2.1% as poor (Figure 1A). Regarding the waiting area, the customers rated the comfort and convenience of the waiting area as follows: 17.1% as excellent, 28% as very as good, 26.7% as good, 18.2% as fair, and 10% as poor (Figure 1B). Furthermore, 10.7% of the respondents ranked the waiting time for services at the pharmacy as very satisfied, 37% satisfied, 40.8% fairly satisfied, and 11.5% not satisfied (Figure 1C). Waiting time was described as the time between the beginning of soliciting pharmacy services and departure from the pharmacy.

### 3.4. Reasons for Visiting the Community Pharmacy

**Figure 2:**
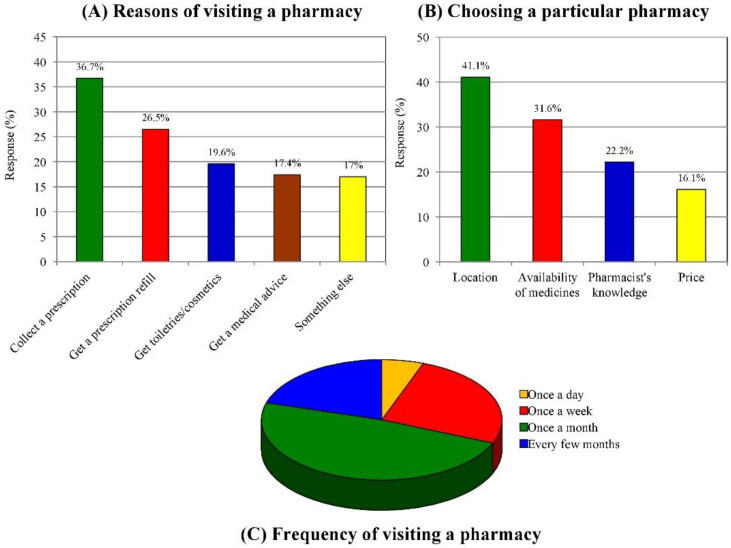
Reasons for visiting the community pharmacy (**A**). Factors contributing to choosing a particular community pharmacy (**B**). Frequency of visiting a pharmacy (**C**).

The participants were asked about their primary reasons for visiting a pharmacy. Most respondents (36.7%) stated that they had visited a pharmacy to purchase their medicines (Figure 2A). Other reasons for visiting a community pharmacy included getting a prescription refill (26.5%), buying toiletries or cosmetics (19.6%), getting medical advice (17.4%), and miscellaneous (17%). We asked the customers about their reasons for visiting a particular community pharmacy; the main reason was the location of the pharmacy (41.1%), as shown in Figure 2B. This included the pharmacy being close to their home or the doctor’s clinic or medical center. Other reasons for patronizing a particular pharmacy were the availability of medicines (31.6%), pharmacist’s knowledge (22.2%), and the price of medicines (16.1%). A total of 483 (48.3%) participants visited the pharmacy once a month, while 257 (25.7%) visited once a week; 56 (5.6 %) consumers visited the pharmacy more than once a day, and 204 (20.4%) visited every few months (Figure 2C).

### 3.5. Image of Community Pharmacists among Customers

**Figure 3:**
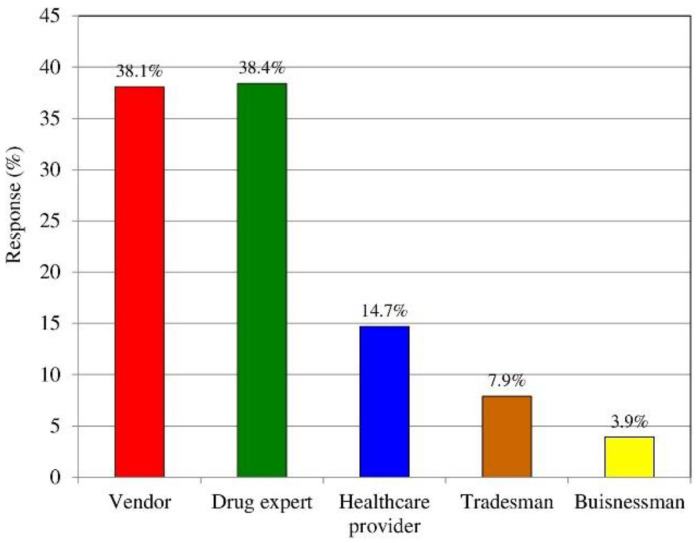
The image of the community pharmacists among customers.

The role of pharmacists of merely dispensing and supplying medicines to customers is evolving to include the provision of a more patient-centered service. We explored the opinions of customers about the general image of their community pharmacist. Out of 1000 participants, 381 (38.1%) viewed the community pharmacist as a vendor of medicines, 384 (38.4%) as a drug expert, 147 (14.7%) as a health care provider, 79 (7.9%) as a tradesman, and 39 (3.9%) as a businessman, as shown in Figure 3. Although pharmacists are deemed to be experts on drugs, they were not perceived as such by many of the respondents.

### 3.6. Customers’ Views on the Availability and Competency of Community Pharmacist 

Most respondents (81.5%) emphasized that community pharmacists are available in the pharmacy, and 80.7% said that they were treated with great respect and politeness by the pharmacist, as shown in Table 3. About 56.7% stated that the pharmacist spent enough time with them, and 71.7% said that the pharmacist was willing to answer their questions clearly. About 71.3% of respondents placed a high level of trust in pharmacists to provide pharmaceutical care and solve medication-related problems. Regarding promptness of the service provided by the pharmacist, they were happy with the way it was offered and rated the service as follows: (31%) excellent, (36.5%) very good, (22.3%) good, (8%) fair, and (2.2%) poor (data not shown).

### 3.7. Views on Societal Acceptance of the Community Pharmacist as a Health Care Provider

**Figure 4:**
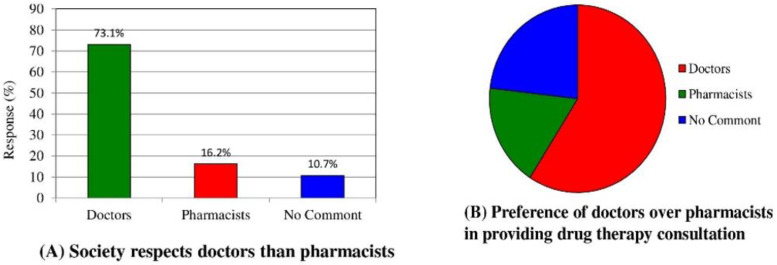
Perception of pharmacists. Customers’ views on societal respect of pharmacists (**A**). Customers’ preferences of doctor or pharmacist for the provision of drug therapy consultation (**B**).

When the customers were asked about their perception of the pharmacist’s job as compared to doctors, most (73.1%) stated that society respects doctors more than pharmacists (16.2%), as shown in Figure 4A. About 59.1% of respondents preferred doctors to pharmacists for the provision of drug therapy consultation (Figure 4B).

### 3.8. Customers’ Views on the Clinical Roles of Community Pharmacists

Community pharmacists are the health care professionals which are most accessible to the public; they play an essential role in educating individuals regarding drug therapy and health issues. The community pharmacy setting is a platform for pharmacists to project themselves to the public as health care providers, and to participate in changing the image of the pharmacist as a mere vendor of medication. This part of the survey comprised four questions about the services offered by pharmacists, such as collecting information about medical conditions and medications, health screening tests (such as measuring blood pressure, blood sugar, temperature, weight), and giving information about lifestyle and health issues. When the respondents were questioned about these roles, they felt that pharmacists had a role to play in terms of providing information about their disease status and medications, as well as health screening services (Table 4). In response to a question on whether pharmacists collected information on current medical conditions from the customers when preparing prescription services, 49.1% answered “Yes” and 35.9% “No”. Regarding the collection of information on current medications used by the customers, 45.5% responded with “Yes” and 40% with “No”. On the topic of general lifestyle and health issues, 31.5% said that pharmacists supplied relevant information about lifestyle (diet, smoking, and physical exercise) while 58.2% said they did not receive any such information. When questioned about whether pharmacists measured blood pressure, blood sugar, temperature and weight, only 24.3% of respondents answered “Yes”, while 64.2% answered “No”. This indicates that the public may be unaware of the availability of such services in community pharmacies, and thus, they go to doctors and hospitals for screening tests.

### 3.9. Opinions of Customers on the Role of Pharmacists in the Provision of Information on the Use of Medicines

This part of the survey comprised six questions on drug costs, drug alternatives, selecting over-the-counter (OTC) drugs, solving drug-related problems, and disposing of unused drugs. When the customers were questioned about their choices of medicines, costs, and drug-related problems, we noted that the answers were variable, as shown in Table 4. Most respondents (72.8%) stated that pharmacists had given them clear instructions on the use of their medications. About 36.9% sought advice on avoiding unnecessary costs related to prescriptions; 77.4% received advice on drug alternatives, 66.2% received advice on selecting over-the-counter medicines, 40.4% received advice on solving drug-related problems (such as side effects, drug interactions, and compliance), and 31.3% received advice on disposing of unused medicines.

### 3.10. Buying Medicines over the Internet and Home Delivery of Medicines

**Figure 5:**
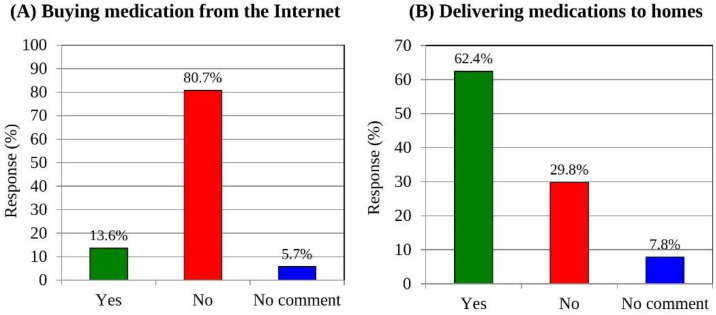
Categorical distribution of respondents’ responses to question on buying medicines over the Internet (**A**) and home delivery of medicines (**B**).

The Internet offers customers a convenient way of purchasing medicines, but this should be approached with great caution. Some websites sell prescription and over-the-counter drugs that may not be safe to use and could put people’s health at risk. When customers were asked about their experiences and willingness to buy pharmaceutical drugs over the Internet, the vast majority (80.7%) answered “No”, with 13.6% answering “Yes” (Figure 5A). On the other hand, a higher percentage of customers (62.4%) agreed to order medicines for home delivery from a pharmacy (Figure 5B).

### 3.11. Satisfaction of Customers with Their Community Pharmacist and Pharmacy Services

**Figure 6:**
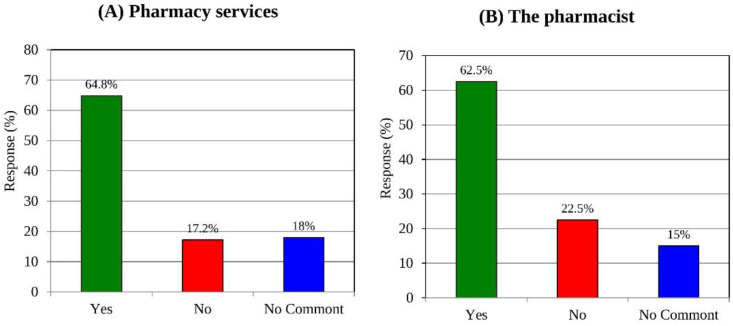
Categorical distribution of respondents’ level of satisfaction with the pharmacy services (**A**) and their community pharmacist (**B**).

Patient satisfaction is used as a measure of the quality of service in the medical and pharmacy sectors. Lastly, we asked the customers about their general level of satisfaction with the roles of community pharmacists and the pharmacy services offered. Most of the respondents were generally satisfied with the role of their community pharmacist (62.5%) and the pharmacy services offered by pharmacy staff (64.8%) (see Figure 6A,B).

## 4. Discussion

In most countries of the Middle East, community pharmacists taking a business-oriented approach and placing profit before customers’ needs perceive giving advice regarding the correct use of medications as a waste of time and something that does not directly involve additional financial remuneration; therefore, they tend to devote less time to patients [31,32]. This cross-sectional study was undertaken in Al-Madinah Al-Munawwarah, Saudi Arabia, to evaluate the perception of customers toward community pharmacies, the roles of community pharmacists, and pharmacy services. Data from a survey of 1000 customers (539 females, 461 males) concerning the views of the customers on community pharmacists and pharmacy services are presented. The study respondents had positive perception toward community pharmacists and pharmacy services. An evaluation of the influence of patient demographics on the level of satisfaction showed that patient satisfaction level was affected by several factors such as the age and educational level of the customer. A higher level of satisfaction with pharmacy services was noted among females than males. The reported higher satisfaction among female respondents was attributable to the emotional buildup of females and their willingness to receive information about their medication from the pharmacist [25,33,34]. Satisfaction with community pharmacy services was also greatly dependent on several factors such as the location of the pharmacy and the promptness of the services (41.11%), the availability of medicines (31.6%), the pharmacist’s knowledge (22.2%), and prices (16.1%). There are many reasons for visiting a pharmacy, including the need to purchase medicines (36.1%) recommended by doctors and to refill prescriptions (26.5%).

Customers’ views about the amenities and accessibility of pharmacies were also explored in the present study. The examples included in our study were the cleanliness and availability of waiting areas, as well as waiting time, which considerably influenced satisfaction levels with a particular pharmacy. In general, most of the participants ranked all these aspects as good. Consultation services and waiting time were considered key factors that could influence patient satisfaction with the delivery of health care services [35,36]. Our study revealed that the respondents rated the waiting areas as either very good (28%) or good (26.7%). More than half of the surveyed customers (52.3%) were satisfied with the waiting time prior to receiving a consultation. Patients who are made to wait longer than expected were less likely to be satisfied with the health care service [37]. Anderson et al. [38] warned that the combination of short consultation times and long waiting times is toxic in terms of patient satisfaction, and that this must be avoided by health care providers. Several studies have examined the concept of privacy and confidentiality in pharmacies in terms of product service, conversations in the pharmacy, the exchange of medical information, and medications purchased [39,40,41]. Pharmacy staff should be discreet when calling out patient names and providing counseling or screening services [40]. In our study, only 63.9% of respondents stated that a lack of privacy at the pharmacy was a barrier to seeking help from community pharmacists. This may be due to insufficient space in the pharmacies themselves and the lack of a private area for consultation in the majority of cases. Saudis feel comfortable seeking advice from their pharmacist despite the possible lack of privacy in pharmacies [23]. A study in the Netherlands reported that a lack of pharmacy privacy led to reluctance among customers to ask questions [42]. There is increased concern among customers about the level of privacy and utilization of community pharmacy services. Therefore, a need to regulate community pharmacy ‘spaces’ to ensure privacy is justified, to allow pharmacists to make use of their knowledge about medicines and health conditions and to make informed decisions regarding treatment options [43].

Traditionally, people believe in the abilities and skills of physicians in terms of providing drug therapy consultations; therefore, they approach physicians when they have drug queries. In this study, only 40.7% of respondents believed community pharmacists to be incompetent in providing them with effective medications or consultations when facing a drug-related problem. A similar study by El Hajj et al. [24] demonstrated that 70% of participants did not consider pharmacists to be capable of performing proper screening and monitoring for specific diseases. Wazaify et al. [26] also reported that most participants (62.7%) would seek advice from a pharmacist only when the condition was not serious enough to visit the doctor. Therefore, it can be said that the Saudi community needs awareness programs and informative campaigns to educate the public about the roles and abilities of community pharmacists in terms of providing patient care and strengthening patient–pharmacist relationships.

As per the response to our questionnaire, although customers considered pharmacists to be experts on drugs (38.4%), unfortunately, they were not perceived as health care providers (14.7%). Rather, study participants viewed pharmacists as mere vendors of medicines (38%) rather than health care professionals. There were some beliefs among customers that pharmacists are more concerned with the business side of the profession than with its health aspect. This result is relatively different when compared to a study by El Hajj et al. [24], where 44% of patients believed that pharmacists demonstrate a good balance between health and business matters. Hargie et al. [44] measured consumer perceptions of, and attitudes toward, community pharmacy services using a communication audit technique, and found that community pharmacists were perceived to be purely business-minded individuals by 32% of respondents; 26% saw them as health-oriented people, and 42% saw them as both health- and business-oriented people. Most participants in this study felt that, although pharmacists ask questions about current medications customers are using, as well as the existence of other medical conditions when preparing the prescription, they do not provide customers with thorough medication counseling and advice on achieving a healthy lifestyle or on medication use. It is well-accepted that community pharmacists in Saudi Arabia, when dispensing medications, consider patients to be well-informed about their medical conditions by their physicians. [24,26,45]

Patient satisfaction is one of the indicators of the quality of medical and pharmacy services provided in health care settings. Several studies have investigated patient satisfaction and attitudes toward community pharmacy services [24,43,46,47,48,49,50,51,52,53,54]. These studies revealed that satisfaction with community pharmacy services was greatly dependent on several factors, including the location of the community pharmacy, the promptness of services, and the pharmacist’s knowledge. In developed countries, patient satisfaction is a key factor in quality assessments of the health care system [44,47], whereas in developing countries, the main factor is accessibility [45,55]. A large proportion of the public in the Middle East does not consider the provision of advice or information to be the primary role of the pharmacists; they feel pharmacists should stick to dispensing medicines [24,26,47,48,56,57]. In our study, customers expressed positive views and satisfaction with community pharmacists and pharmacy services.

Community pharmacists come from different backgrounds and pharmacy curricula. These curricula may not adequately prepare graduates to actively participate in pharmaceutical care. Pharmaceutical organizations and authorities must ensure that community pharmacists are equipped with sufficient clinical skills to deliver appropriate services at community pharmacies. In addition, Saudi community pharmacists should make considerable efforts to raise public awareness about their professional role in terms of the safe use medications and the monitoring and counseling of patients.

### Limitations of the Study

The main limitation of this study was the dearth of open-ended questions, as closed-ended questions limit the respondents to specific response categories that may not include all response possibilities. Furthermore, the findings cannot be applied generally to all contexts in Saudi Arabia. The perspectives may be different among other community pharmacies in different areas, or in settings with different working hours or workloads of the pharmacist. Additionally, other factors in terms of experiences and contact with pharmacists may be different. Additionally, the female to male ratio of respondents (1:0.85) was not representative of the gender distribution of the population in Saudi Arabia (1:1.3). Moreover, the educational level of participants was biased toward the highly educated in comparison to the general population; as a result, the findings should be evaluated in light of this constraint. Similarly, the distribution of the participants was biased toward younger age groups, so the findings may not accurately reflect the perceptions of older people. However, the predominance of younger people can be viewed as a strength, because the young will experience and participate in the ongoing reform of the Saudi healthcare system [58].

## 5. Conclusions

The study findings showed that most respondents were generally satisfied with their pharmacists and current pharmacy services. The study amplifies the need to highlight the roles of pharmacists in the community in order for them to be recognized as a health care providers and accepted as an integral partner in the health care profession.

Customers’ opinions were influenced by the availability and knowledge of pharmacists, pharmacy service promptness, pharmacy location, waiting area, knowledge about medications, and counseling. Customers were greatly satisfied with their pharmacists’ professionalism and with the pharmaceutical services they used.

## Figures and Tables

**Table 1:** Demographic Characteristics of Study Respondents.

Public’s Respondents (No. = 1000)	Number (%)
Gender	
a. Male b. Female	461 (46.1) 539 (53.9)
2. Age	
a. <20 years b. 20–39 years c. 40–59 years d. ≥60 years	240 (24) 489 (48.9) 219 (21.9) 52 (5.2)
3. Nationality	
a. Saudi b. Non-Saudi	905 (90.5) 95 (9.5)
4. Marital status	
a. Single b. Married c. Divorced d. Widow	497 (49.7) 443 (44.3) 40 (4) 20 (2)
5. Educational level	
a. College or higher b. High school c. Less than high school d. Illiterate	579 (57.9) 304 (30.4) 79 (7.9) 38 (3.8)
6. Employment status	
a. Full-time b. Part-time c. Not working	319 (31.9) 197 (19.7) 484 (48.4)
7. Income	
a. High b. Reasonable c. Low	163 (16.3) 576 (57.6) 261 (26.1)
8. Health status	
a. Diabetes b. Hypertension c. Asthma d. Cardiac e. Other f. None	128 (12.8) 138 (13.8) 92 (9.2) 34 (3.4) 413 (41.3) 195 (19.5)

**Table 2:** Respondents’ Views on the Community Pharmacy.

Questions	Yes (%)	No (%)	No Comment (%)
Does the community pharmacy have the medicines or appliances you need?	49	34.1	16.9
2. Is there someone in the community pharmacy available to serve you?	74.6	16.9	8.5
3. Is there a place in the community pharmacy where you can speak without being overheard?	25.6	63.9	10.5
4. Could you differentiate between the pharmacist and the nonpharmacist staff in the community pharmacy?	71.5	21.7	6.8
5. Does the community pharmacy have a computer system?	71.7	20.3	8

**Table 3:** Customers’ Views on Community Pharmacists.

Questions	Yes (%)	No (%)	No Comment (%)
1- Is the pharmacist available in the pharmacy at the designated hours?	815 (81.5)	97 (9.7)	88 (8.8)
2- Does the pharmacist deal with you with interest and respect?	807 (80.7)	90 (9)	103 (10.3)
3- Does the pharmacist spend enough time with you?	567 (56.7)	258 (25.8)	175 (17.5)
4- Does the pharmacist answer all your questions?	717 (71.7)	167 (16.7)	116 (11.6)
5- Do you trust the pharmacist’s opinion regarding medication?	713 (71.3)	142 (14.2)	(14.5)

**Table 4:** Responses to Questions on the Clinical Roles of Community Pharmacists.

Questions	Yes (%)	No (%)	No Comment (%)
**A**. Patient and Drug History			
1- Did the pharmacist ask about your medical condition when preparing your prescription?	491 (49.1)	359 (35.9)	150 (15)
2- Did the pharmacist ask about other medications you use when preparing your prescription?	455 (45.5)	400 (40)	145 (14.5)
3- Did the pharmacist provide general advice on healthy lifestyle, smoking and physical exercise?	315 (31.5)	(58.2)	103 (10.3)
4- Did the pharmacist measure/monitor any of the following: blood pressure, blood sugar, temperature or weight?	243 (24.3)	642 (64.2)	115 (11.5)
**B**. Medication Use			
1- Did the pharmacist provide clear instructions about the use of the medications?	728 (72.8)	171 (17.1)	101 (10.1)
2- Did you get the help from the pharmacist to avoid unnecessary costs related to your prescriptions?	369 (36.9)	500 (50)	131 (13.1)
3- Did the pharmacist give you alternatives when the drug you required was not available?	774 (77.4)	160 (16)	66 (6.6)
4- Do you get the help from the pharmacist in the selection of OTC and herbal medicines?	662 (66.2)	248 (24.8)	90 (9)
5- Did the pharmacist address any drug-related problems (side effects, drug interactions, compliance) that concerned you?	407 (40.7)	413 (41.3)	180 (18)
6- Did the pharmacist provide advice on disposing of medicines you no longer need?	313 (31.3)	534 (53.4)	153 (15.3)

## References

[1] Wiedenmayer K., Summers R.B., Mackie C.A., Gous G.A., Everard M. (2006). Developing Pharmacy Practice: A focus on Patient Care.

[2] Penna R.P. (1990). Pharmaceutical care: Pharmacy’s mission for the 1990s. Am. J. Hosp. Pharm..

[3] Beney J., Bero L.A., Bond C. (2000). Expanding the roles of outpatient pharmacists: Effects on health services utilisation, costs, and patient outcomes. Cochrane Database Syst. Rev..

[4] Hermansen C.J., Wilderholt J.B. (2001). Pharmacist-patient relationship development in an ambulatory clinic setting. Health Commun..

[5] Rasheed M.K., Alqasoumi A., Hasan S.S., Babar Z.U.D. (2020). The community pharmacy practice change towards patient-centered care in Saudi Arabia: A qualitative perspective. J. Pharm. Policy Pract..

[6] Schommer J.C. (1994). Effects of interrole congruence on pharmacist–patient communication. J. Health Commun..

[7] Moorman C., Deshpande R., Zaltman G. (1993). Factors affecting trust in marketing relationships. J. Mark..

[8] Mayer R.C., Davis J.H., Schoorman F.D. (1995). An integrative model of organizational trust. Acad Manag. Rev..

[9] Schommer J.C., Kucukarslan S.N. (1997). Measuring patient satisfaction with pharmaceutical services. Am. J. Health-Syst. Pharm..

[10] Kassam R., Collins J.B., Berkowitz J. (2009). Developing anchored measures of patient satisfaction with pharmaceutical care delivery: Experiences versus expectations. Patient Prefer. Adherence.

[11] Larson L.N., Rovers J.P., MacKeigan L.D. (2002). Patient satisfaction with pharmaceutical care: Update of a validated instrument. J. Am. Pharm. Assoc..

[12] Ried L.D., Wang F., Young H., Awiphan R. (1999). Patients’ satisfaction and their perception of the pharmacist. J. Am. Pharm. Assoc..

[13] Mira J.J., Aranaz J. (2000). Patient satisfaction as an outcome measure in health care. Med. Clin..

[14] Hall J., Dornan M. (1998). Meta-analysis of satisfaction with medical care: Description of research domain and analysis of overall satisfaction levels. Soc. Sci. Med..

[15] Ikegami N., Kawakita H. (1987). Patient satisfaction and hospital management policy. J. Jpn. Hosp. Assoc..

[16] NaikPanvelkar P., Saini B., Armour C. (2009). Measurement of Patient Satisfaction with Community Pharmacy Services: A Review. Pharm. World. Sci..

[17] Pascoe G.C. (1983). Patient satisfaction in primary health care: A literature review and analysis. Eval. Program Plann..

[18] Marquis M.S., Davies A.R., Ware J.E. (1983). Patient satisfaction and change in medical care provider: A longitudinal study. Med. Care.

[19] Zastowny T.R., Roghmann K.J., Cafferata G.L. (1989). Patient satisfaction and the use of health services: Explorations in causality. Med. Care.

[20] Gourley G.K., Gourley D.R., La Monica Rigolosi E., Reed P., Solomon D.K., Washington E. (2001). Development and validation of the pharmaceutical care satisfaction questionnaire. Am. J. Manag. Care.

[21] Hasan S., Sulieman H., Stewart K., Chapman C.B., Hasan M.Y., Kong D.C. (2013). Assessing patient satisfaction with community pharmacy in the UAE using a newly-validated tool. Res. Soc. Adm. Pharm..

[22] Khdour M.R., Hallak H.O. (2012). Societal perspectives on community pharmacy services in WestBank—Palestine. Pharm. Pract..

[23] Bawazir S.A. (2004). Consumer attitudes towards community pharmacy services in Saudi Arabia. Int. J. Pharm. Pract..

[24] El Hajj M.S., Salem S., Mansoor H. (2011). Public’s attitudes towards community pharmacy in Qatar: A pilot study. Patient Prefer. Adherence.

[25] Al-Arifi M.N. (2012). Patients’ perception, views and satisfaction with pharmacists role as health care provider in community pharmacy setting at Riyadh, Saudi Arabia. Saudi Pharm. J..

[26] Wazaify M., Al-Bsoul-Younes A., Abu-Gharbieh E., Tahaineh L. (2008). Societal perspectives on the role of community pharmacists and over-the-counter drugs in Jordan. Pharm. World Sci..

[27] Al Akshar S., Metwaly Z., Shamssain M. (2014). Patients’ perceptions of community pharmacy practice in UAE: An overview. IOSR J. Pharm..

[28] Aminuddin Y., Parilah M.S., Jacklyn J. (2017). Customer’s expectation, perception and satisfaction with service quality of a fitness center in Malaysia. Inter. J. Phys. Educ. Sports Health.

[29] Ismail A., Gan Y.N., Ahmad N. (2020). Factors associated with patient satisfaction towards pharmacy services among out-patients attending public health clinics: Questionnaire development and its application. PLoS ONE.

[30] Gidman W., Cowley J. (2013). A qualitative exploration of opinions on the community pharmacists’ role amongst the general public in Scotland. Int. J. Pharm. Pract..

[31] Morrow N., Owen H., Woodman C. (1993). Consumer perceptions of and attitudes to the advice-giving role of community pharmacists. Pharm. J..

[32] Al Ruthia Y., Alsenaidy M.A., Alrabiah H.K., Al Muhaisen A., Alshehri M. (2018). The status of licensed pharmacy workforce in Saudi Arabia: A 2030 economic vision perspective. Hum. Resour. Health.

[33] Almohammed O.A., Alsanea S. (2021). Public Perception and Attitude toward Community Pharmacists in Saudi Arabia. Saudi J. Health Syst. Res..

[34] Ghattas D., Al-Abdallah G. (2020). Factors affecting customers selection of community pharmacies: The mediating effect of branded pharmacies and the moderating effect of demographics. Manag. Sci. Lett..

[35] Thompson D.A., Yarnold P.R. (1995). Relating patient satisfaction to waiting time perceptions and expectations: The disconfirmation paradigm. Acad. Emerg. Med..

[36] Sanmartin C., Berthelot J.M., Mcintosh C.N. (2007). Determinants of Unacceptable waiting times for specialized services in Canada. Health Policy.

[37] Thompson D.A., Yarnold P.R., Williams D.R., Adams S.L. (1996). Effects of actual waiting time, perceived waiting time, information delivery and expressive quality on patient satisfaction in the emergency department. Ann. Emerg. Med..

[38] Anderson R.T., Camacho F.T., Balkrishnan R. (2007). Willing to wait? the influence of patient wait time on satisfaction with primary care. BMC Health Serv. Res..

[39] Alsuhebany N., Alfehaid L., Almodaimegh H., Albekairy A., Alharbi S. (2019). Attitude and perception of physicians and nurses toward the role of clinical pharmacists in Riyadh, Saudi Arabia: A qualitative study. SAGE Open Nurs..

[40] Hattingh H., Knox K., Fejzic J., McConnell D., Fowler J.L., Mey A., Kelly F., Wheeler A.J. (2015). Privacy and confidentiality: Perspectives of mental health consumers and carers in pharmacy settings. Int. J. Pharm. Pract..

[41] Taylor J., Krska J., Mackridge A. (2012). A community pharmacy-based cardiovascular screening service: Views of service users and the public. Int. J. Pharm. Pract..

[42] Pronk M., Blom A., Jonkers R., Bakker A. (2003). Evaluation of patient opinions in a pharmacy-level intervention study. Int. J. Pharm. Pract..

[43] Khogah H. (2019). Privacy level in private community pharmacies in Saudi Arabia: A simulated client survey. Pharmacol. Pharm..

[44] Hargie O., Morrow N., Woodman C. (1992). Consumer perceptions of and attitudes to community pharmacy services. Pharm. J..

[45] El Hajj M.S., Mekkawi R., Elkaffash R., Saleh R., El Awaisi A., Wilbur K. (2021). Public attitudes towards community pharmacy in Arabic speaking Middle Eastern countries: A systematic review. Res. Soc. Adm. Pharm..

[46] Gold M., Woodridge J. (1995). Surveying consumer satisfaction to assess managed care quality: Current practices. Health Care Financ. Rev..

[47] Khayyat S., Walters P., Whittlesea C., Nazar H. (2021). Patient and public perception and experience of community pharmacy services post-discharge in the UK: A rapid review and qualitative study. BMJ Open.

[48] Mera J.A. (2002). A review of the “welfare state” and alternative ways of delivering health care. Int. J. Qual. Health Care.

[49] Ibrahim I.R., Al Tukmagi H.F., Wayyes A. (2013). Attitudes of Iraqi society towards the role of community pharmacists. Innov. Pharm..

[50] Wirth F., Taboneb F., Azzopardi L.M., Gauci M., Zarb-Adami M., Serracino-Inglott A. (2010). Consumer perception of the community pharmacist and community pharmacy services in Malta. J Pharm. Health. Serv. Res..

[51] Cavaco A.M., Dias J.P., Bates I.P. (2005). Consumers’ perceptions of community pharmacy in Portugal: A qualitative exploratory study. Pharm. World Sci..

[52] Cerulli J. (2002). Patients perceptions of independent community pharmacists. J. Am. Pharm. Assoc..

[53] Farris K.B., Stenton S.B., Samnani M., Samycia D. (2000). How satisfied are your patients?. Can. Pharm. J..

[54] Kamei M., Teshima K., Fukushima N., Nakamura T. (2001). Investigation of patients demand for community pharmacies: Relationship between pharmacy services and patient satisfaction. Yakugaku Zasshi.

[55] Kashif K., Qaiser I., Sajjad H., Muhammad A., Rabia I., Fahad S. (2020). Public’ Perception, awareness, expectations and experiences towards the role of community pharmacists in Quetta City, Pakistan. J. Pharm. Pract. Community Med..

[56] Matar M.S., Eljanzoury A.M., Musa S.I., Abdulwahaab M.A., Mustafa A.A., Yousef B.A., Badi S. (2021). Evaluation of counseling services provided by community pharmacists and patients’ satisfaction toward their services: A cross-sectional survey from Sudan. Curr. Med. Issues.

[57] Alssageer M.A., Hassan A.O., Rajab M.O. (2021). Consumers view, expectation and satisfaction with community pharmacy services. Mediterr. J. Pharm. Pharm. Sci..

[58] Alhaddad M. (2019). Youth experience with community pharmacy services and their perceptions toward implementation of medication therapy management services by community pharmacists in the western region of Saudi Arabia. Innov. Regul. Sci..

